# Upregulation of FOXM1 induces genomic instability in human epidermal keratinocytes

**DOI:** 10.1186/1476-4598-9-45

**Published:** 2010-02-26

**Authors:** Muy-Teck Teh, Emilios Gemenetzidis, Tracy Chaplin, Bryan D Young, Michael P Philpott

**Affiliations:** 1 Centre for Clinical and Diagnostic Oral Sciences, Institute of Dentistry, Barts and the London School of Medicine and Dentistry, Queen Mary University of London, Turner Street, London E1 2AD, UK; 2 Cancer Genomics Group, Medical Oncology Centre, Barts and the London School of Medicine, Queen Mary University of London, Charterhouse Square, London EC1M 6BQ, UK; 3 Centre for Cutaneous Research, Blizard Institute of Cell and Molecular Science, Barts and the London School of Medicine and Dentistry, Queen Mary University of London, Turner Street, London E1 2AD, UK

## Abstract

**Background:**

The human cell cycle transcription factor FOXM1 is known to play a key role in regulating timely mitotic progression and accurate chromosomal segregation during cell division. Deregulation of FOXM1 has been linked to a majority of human cancers. We previously showed that FOXM1 was upregulated in basal cell carcinoma and recently reported that upregulation of FOXM1 precedes malignancy in a number of solid human cancer types including oral, oesophagus, lung, breast, kidney, bladder and uterus. This indicates that upregulation of FOXM1 may be an early molecular signal required for aberrant cell cycle and cancer initiation.

**Results:**

The present study investigated the putative early mechanism of UVB and FOXM1 in skin cancer initiation. We have demonstrated that UVB dose-dependently increased FOXM1 protein levels through protein stabilisation and accumulation rather than de novo mRNA expression in human epidermal keratinocytes. FOXM1 upregulation in primary human keratinocytes triggered pro-apoptotic/DNA-damage checkpoint response genes such as p21, p38 MAPK, p53 and PARP, however, without causing significant cell cycle arrest or cell death. Using a high-resolution Affymetrix genome-wide single nucleotide polymorphism (SNP) mapping technique, we provided the evidence that FOXM1 upregulation in epidermal keratinocytes is sufficient to induce genomic instability, in the form of loss of heterozygosity (LOH) and copy number variations (CNV). FOXM1-induced genomic instability was significantly enhanced and accumulated with increasing cell passage and this instability was increased even further upon exposure to UVB resulting in whole chromosomal gain (7p21.3-7q36.3) and segmental LOH (6q25.1-6q25.3).

**Conclusion:**

We hypothesise that prolonged and repeated UVB exposure selects for skin cells bearing stable FOXM1 protein causes aberrant cell cycle checkpoint thereby allowing ectopic cell cycle entry and subsequent genomic instability. The aberrant upregulation of FOXM1 serves as a 'first hit' where cells acquire genomic instability which in turn predisposes cells to a 'second hit' whereby DNA-damage checkpoint response (eg. p53 or p16) is abolished to allow damaged cells to proliferate and accumulate genetic aberrations/mutations required for cancer initiation.

## Background

The forkhead box (FOX) transcription factors have been shown to regulate cell growth, proliferation, differentiation, longevity and transformation and exhibit a diverse range of functions during embryonic development and adult tissue homeostasis [reviewed in [1]]. FOXM1-null mouse embryos were neonatal lethal as a result of the development of polyploid cardiomyocytes and hepatocytes, highlighting the role of FOXM1 in mitotic division [2]. More recently a study using transgenic/knockout mouse embryonic fibroblasts and human osteosarcoma cells (U2OS) has shown that FOXM1, regulates expression of a large array of G2/M-specific genes, such as Plk1, Cyclin B2, Nek2 and CENP-F, and plays an important role in maintenance of chromosomal segregation and genomic stability [3].

A key intrinsic mechanism that determines cell survival and apoptosis is the ability to detect and respond to genotoxic insults such as chemical carcinogens, ultraviolet or ionising irradiation. Failure to regulate DNA damage response checkpoints and subsequent genomic stability in cells often leads to tumourigenesis [4]. The forkhead protein FOXO3a has been shown to play a role in both DNA repair pathways and cell cycle checkpoint in response to DNA damage [5]. Moreover, it has recently been reported that FOXO3a can be modulated by oncogenes such as MUC1 causing increased DNA repair and enhanced cell survival in response to oxidative stress [6] and recently FOXM1 was shown in a cancer cell line to stimulate DNA repair genes following genotoxic stress [7].

Basal cell carcinoma (BCC) accounts for up to 20% of all Caucasian carcinomas. We were the first to establish a link between FOXM1 and tumourigenesis when we demonstrated that FOXM1 is upregulated in BCC [8]. Since then, FOXM1 has been implicated in the majority of solid human cancers [reviewed in [9]]. We recently showed that FOXM1 expression precedes malignancy in a number of solid human cancer types including oral, oesophagus, lung, breast, kidney, bladder and uterus indicating its pivotal role in cancer initiation [10]. The present study investigated the putative early mechanism of UVB and FOXM1 in skin cancer initiation. We have used a high efficiency long-term retroviral transduction system to express exogenous FOXM1B in both immortal and primary normal human epidermal keratinocytes (NHEK) to replicate oncogenic levels found in cancer cells. Using Affymetrix SNP microarray to profile genomic instability we show that upregulation of FOXM1B in epidermal keratinocytes results in genomic instability and that this is augmented by UVB, a major aetiological factor in BCC.

## Methods

### Cell culture

Primary NHEK and N/TERT cells [11] were cultured in a low calcium (0.06 mM) EpiLife^® ^keratinocyte growth medium (#M-EPI-500-CA; Cascade Biologics, TCS CellWorks Ltd., Buckinghamshire, UK.) with growth supplements (HKGS, #ZHS-8943; Cascade Biologics). Cells were grown at 37°C in a humidified atmosphere of either 5% (for EpiLife) or 10% (for DMEM) CO_2_/95% air.

### Real-time quantitative PCR

Poly-A^+ ^mRNA extraction, reverse transcription and real-time absolute quantitative PCR (qPCR) protocols are MIQE compliant [12] and were performed as described previously [10] using a LightCycler LC480 instrument (Roche Diagnostic). EGFP primers GFP-F2, 5'-TGGCCGACAAGCAGAAGAAC-3' and GFP-R2, 5'-CTTCTCGTTGGGGTCTTTGCTC-3' were used to quantify the levels of viral transduction by measuring the EGFP transgene (will detect both EGFP and EGFP-FOXM1B transgenes) copy number present in the genomic DNA of transduced cells. Viral supernatant were titrated to achieve FOXM1B mRNA expression levels of around 5 to 10-fold upregulation over normal NHEK. This level of FOXM1B upregulation was found in various keratinocyte cancer cell lines such as UK1 and SCC15 [10]. Statistical analysis was performed using the GraphPad InStat software (V2.04a, GraphPad Software, San Diego, CA) for Student's t-test analysis.

### Retroviral transduction and FOXM1 reporter assay

Retroviral supernatant and transduction procedures were performed as reported previously [8,10,13]. Equal levels of EGFP and EGFP-FOXM1B expression were achieved by serial retroviral supernatant titration experiment and subsequently EGFP copy number confirmed by qPCR using genomic DNA extracted from transduced cells.

### UVB irradiation, FACS analysis and cell viability assay

Semi-confluent cells in 10 cm^2 ^dishes were rinsed and covered with a thin layer of PBS (2 ml) for UVB irradiation (UVP CL-1000 Ultraviolet Cross-Linker with F8T5 bulbs) with lids removed during irradiation. UVB-dose titration experiment was performed to determine an intermediate dose that produces partial apoptosis at 24 hours for primary NHEK, N/TERT and HaCaT were found to be 10-20 mJ/cm^2^. For FACS-propidium iodide analysis, culture medium was centrifuged together with trypsinised cells to collect all cells including detached cells. Each cell pellet was resuspended in 100 μl PBS and 1 ml 70% ethanol was then added and FACS performed.

### Western blotting

Protein samples were separated on SDS-polyacrylamide gels and transferred to nitrocellulose membrane (Hybon-C Extra, Amersham Pharmacia) according to standard protocols. Antibodies used were rabbit polyclonal anti-FOXM1 (K-19, Santa Cruz), mouse monoclonal anti-p21 (Santa Cruz), rabbit polyclonal anti-phospho-p53 (Ser 15) (Cell Signaling), rabbit polyclonal anti-GFP (Abcam), rabbit polyclonal anti-phospho-p38 MAPK (Cell Signaling), rabbit polyclonal anti-PARP (Cell Signaling) and mouse monoclonal anti-GAPDH (Abcam).

### Time-lapse Fluorescence microscopy and digital pixel densitometry

To synchronize cells at G1/S phase by double thymidine block, 2 × 10^5 ^cells were plated in 6 cm dishes. When cells reached 40-50% confluence, 2 mM thymidine was added and incubated for 16 hours in EpiLife medium without growth supplements. Cells were then washed twice with PBS and grown in complete medium for another 9 hours. Thereafter, cells were treated again with 2 mM thymidine in growth supplement-free EpiLife medium for another 12-16 hours. Release from the second thymidine block was performed by washing twice with PBS and replacing with complete EpiLife growth medium when cells were exposed to UVB (0 hour). Time-lapse microscopy was performed at 20 minute intervals for 72 hours where n = 6 fluorescence and brightfield images were recorded from each test well at each time point using the Metamorph software linked to a fluorescence microscope (Nikon Eclipse TE200S) equipped with a temperature-controlled humidified chamber with 5% CO_2_/95% atmospheric air. Digital pixel densitometry was performed as described previously [10].

### SNP Microarray Mapping Assay

Genomic DNA (gDNA) samples were processed for SNP Mapping 10K (V2.0) XbaI Assay protocol (Affymetrix Inc., Santa Clara, CA) array analysis as described previously [10,14]. LOH and LOH likelihood were analyzed using Affymetrix Copy Number Analysis Tool software (CNAT, version 4) [15] and CNV obtained using Copy Number Analyzer for GeneChip (CNAG, version 2) [16]. The mean ± SEM of SNP call rates for all samples (n = 21 chips) used in this study was 96.77% ± 0.00484. Grouping criteria of 10 adjacent SNPs were used to identify CNV and LOH loci and putative genes within or adjacent these loci were identified using IdeogramBrowser (version 0.20.0) [17] based on NCBI Human Genome Assembly (Built 36.2 database). Raw SNP genotype data files have been deposited at the NCBI's Gene Expression Omnibus database [GEO:GSE16937].

## Results

### UVB dose-dependently elevates FOXM1 protein stability and accumulation in keratinocytes

We have recently shown that upregulation of FOXM1B in oral keratinocytes induced genomic instability and that this was augmented by nicotine [10]. Because FOXM1B is upregulated in BCC [8] and since ultraviolet B (UVB, 290-320 nm) is known to be one of the etiological factors in BCC formation [18] we investigated the effects of FOXM1B expression on human keratinocytes and their response to UVB.

We have used retrovirus-mediated transduction of EGFP-FOXM1B fusion protein under the control of a constitutive CMV promoter, in both primary normal human epidermal keratinocytes (NHEK) and the hTERT-immortalised keratinocyte cell line N/TERT which retains normal epidermal keratinocyte differentiation in organotypical cultures and has functional p53/p21 [11]. The system of FOXM1 over-expression used herein has been previously used and confirmed to produce transcriptionally active FOXM1 protein [8,10]. Furthermore, we have previously shown that primary human skin keratinocytes retain active FOXM1 protein which binds to and activates the promoter of CEP55 gene [10]. Fluorescence activated cell sorting (PI-FACS) with propidium iodide of non-irradiated keratinocytes showed no obvious change in cell cycle of FOXM1B overexpressing keratinocytes (see below) and is consistent with the lack of cellular phenotype previously reported [19,20].

**Figure 1:**
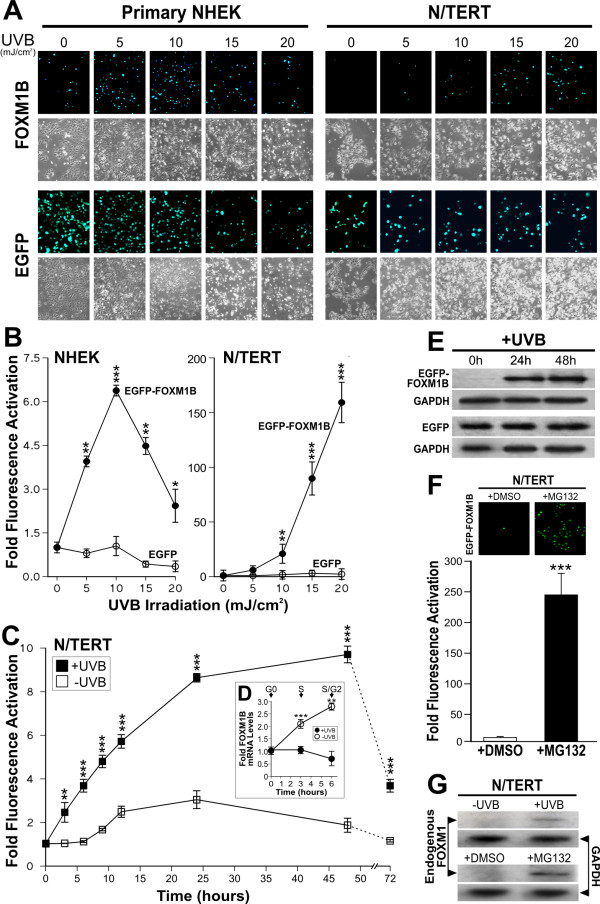
**UVB dose-dependently stabilised FOXM1B protein expression through inhibition of proteolysis**. (A) Fluorescence and phase-contrast microscopy of EGFP or EGFP-FOXM1B transduced cells 24 hours following UVB exposure. (B) Digital densitometry of fluorescence micrographs in (A) as mean ± s.e.m. (n = 3) fold fluorescence activation over control un-irradiated cells. Statistical significance levels: *(P < 0.05), **(P < 0.01) and ***(P < 0.001). (C) Time-lapse fluorescence microscopy of EGFP-FOXM1-transduced N/TERT cells following no-exposure (controls) or UVB irradiation. Each point represents mean ± s.e.m. (n = 6) fold fluorescence activation over control un-irradiated cells at time 0 hour. (D) qPCR showing no change in FOXM1B mRNA during the first 6 hours following UVB exposure. Control non-irradiated cells showed significant increase in FOXM1B mRNA, corresponding cell cycle phases were verified by FACS analyses. (E) Immunoblots showing increased in EGFP-FOXM1 protein levels (using GFP antibody) at 24 and 48 hours following UVB exposure. EGFP-expressing N/TERT showed no change in EGFP protein levels at all time points. GAPDH showed sample loading density in each blot. (F) Proteasomal proteolysis inhibition by MG132 prevented protein degradation leading to stabilisation of FOXM1B proteins. N/TERT cells transduced with EGFP-FOXM1B were treated with either vehicle (0.001% DMSO) or MG132 (1 μM; 24 hours). Fluorescence densitometry showed over 95% ***(P < 0.001) re-activation of EGFP-FOXM1B following MG132 treatment. (G) Immunoblots showing FOXM1 protein stabilisation by UVB and MG132.

However, upon UVB irradiation, we found that UVB dose-dependently increased the expression of EGFP-FOXM1B in both transduced primary NHEK (>6.3-fold increased over non-irradiated cells) and N/TERT (>160-fold; Fig. 1A-B). The dramatic induction of EGFP-FOXM1B in N/TERT compared to primary NHEK may be due to the lower baseline EGFP-FOXM1B expression levels in N/TERT cells compared to primary NHEK prior to UVB exposure (see below) and which may reflect higher turn-over of EGFP-FOXM1B protein in N/TERT. The consistently higher levels of EGFP-FOXM1B in primary NHEK prior to UVB was not due to unequal transduction efficiency because following UVB, both cell types showed over 95% EGFP-FOXM1B re-expression. qPCR analyses showed that gDNA extracted from EGFP and EGFP-FOXM1B transduced cells contain similar levels of EGFP viral transgene indicating that equal viral transduction efficiency was achieved (data not shown). Moreover, UVB did not affect the EGFP protein level in either cell types indicating that the UVB-induced expression of EGFP-FOXM1B was not due to non-specific activation of the CMV promoter.

**Figure 2:**
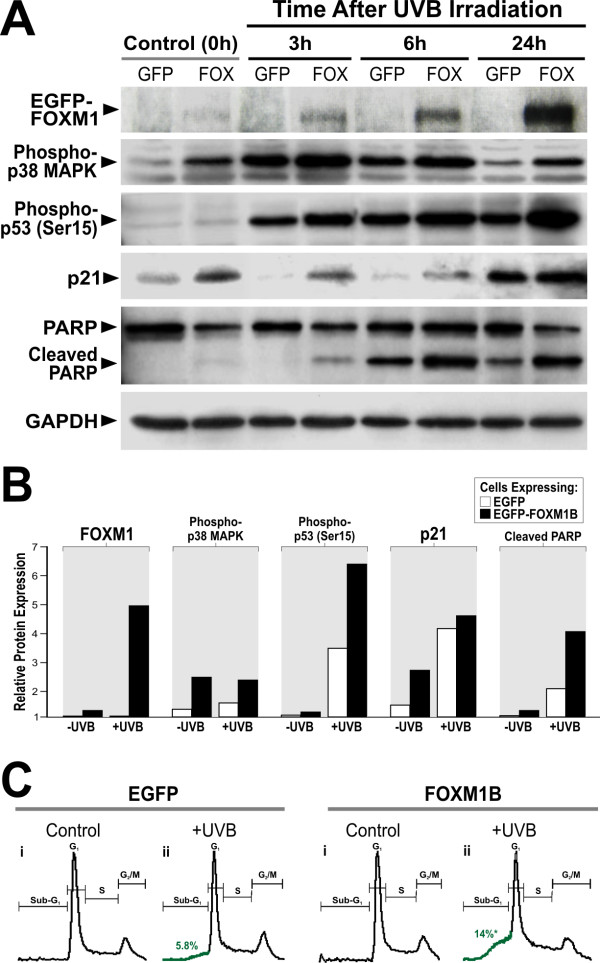
**Upregulation of FOXM1B sensitises cells to UVB-induced apoptosis**. (A) FOXM1B upregulation preferentially activated p21, p38, p53 and increase PARP cleavage in primary NHEK following UVB exposure compared to EGFP controls. Immunoblots of EGFP-FOXM1B (using GFP antibody; EGFP-FOXM1B at ~130 kD and not shown are the EGFP bands which run at ~27kD), phospho-p38 MAPK, phospho-p53 (Ser 15), p21, PARP and GAPDH on primary NHEK transduced with either EGFP (GFP) or EGFP-FOXM1B (FOX). Protein lystates were harvested from cells at time 0 (control un-irradiated), 3, 6 and 24 hours following UVB irradiation as indicated. (B) Digital densitometry graphical representations of data in (A). (C) UVB irradiated FOXM1B-overexpressed cells showed a significant *(P < 0.05) 2.4-fold (5.8% in EGFP cells vs 14% in FOXM1B cells) increased in Sub-G1 population. This result is a representative of 3 independent experiments performed in different occasions using different primary NHEK cells.

To understand how UVB increases FOXM1 protein levels, we used time-lapse fluorescence microscopy to visualise the dynamics of EGFP-FOXM1B protein expression in live N/TERT cells from 0-72 hours following UVB irradiation. All N/TERT cells were synchronised at G1/S phase by double thymidine block prior to the experiment. Cell cycle phases were confirmed by PI-FACS analysis. Non-irradiated EGFP-FOXM1B expressing cells showed increased fluorescence beginning at 8-10 hours and which reached maximum expression levels (~3-fold, Fig. 1C) at 15-25 hours, consistent with the role of FOXM1B in S and G2/M phase expression. In contrast, UVB-irradiated EGFP-FOXM1B expressing cells showed a very rapid increase in fluorescence beginning at 3 hours (~2.5-fold, p < 0.01; Fig. 1C) increasing to over ~8-fold at 24 hours and still remaining high at 48 hours (~9-fold, p < 0.001; Fig. 1C). This pattern of fluorescence expression was consistent with EGFP-FOXM1B protein levels detected by immunoblotting following UVB (Fig. 2A). Using qPCR, mRNA harvested at 0, 3 and 6 hours post UVB showed that FOXM1B mRNA expression was significantly suppressed, whereas, control non-irradiated cells showed rapid increase in FOXM1B expression upon release from growth arrest (Fig. 1D). To confirm that the fluorescence levels correlated with EGFP-FOXM1B protein, we performed immunoblotting using a GFP antibody to determine the level of EGFP and EGFP-FOXM1B protein in cells before and after UVB exposure. In agreement with fluorescence levels, UVB dramatically increased EGFP-FOXM1B protein level 24 and 48 hours after UVB irradiation but had no effect on EGFP alone (Fig. 1E).

In non-irradiated cells, EGFP-FOXM1B protein levels were very low suggesting a rapid turn-over of FOXM1B in cycling cells [21]. EGFP expressing cells did not show fluctuations in protein level before or after UVB exposure (Fig. 1E). Because a significant activation of EGFP-FOXM1B fluorescence was seen as early as 3 hours following UVB irradiation and yet FOXM1B mRNA expression was not activated at this time point (Fig. 1D) suggests that the increase in EGFP-FOXM1B fluorescence was not due to non-specific UVB-induced activation of the CMV promoter. To investigate whether UVB-induced increase in EGFP-FOXM1B fluorescence was due to protein stabilisation, we treated EGFP-FOXM1B expressing N/TERT cells with either vehicle (0.001% DMSO) or a proteasomal proteolysis inhibitor MG132 [22]. We showed that MG132 (1 μM, 24 hours), but not vehicle (0.001% DMSO, 24 hours), significantly increased EGFP-FOXM1B fluorescence level by more than ~240-fold in previously non-irradiated N/TERT cells (Fig. 1F). MG132 did not affect EGFP fluorescence level. The high level of EGFP-FOXM1B fluorescence seen after MG132 treatment indicates that majority of the transduced cells carry the EGFP-FOXM1B transgene (i.e. viral transduction was highly efficient) and that the rapid EGFP-FOXM1B protein turnover could be stabilised by inhibition of proteolysis. Furthermore, inhibition of de novo protein synthesis by cycloheximide treatment (25 μg/ml, 24 hours) did not prevent the accumulation of EGFP-FOXM1B protein following UVB exposure. This is consistent with the qPCR experiments showing that FOXM1B mRNA levels did not increase following UVB exposure (Fig. 1D).

To confirm that endogenous FOXM1 protein was also stabilised by UVB and MG132, a FOXM1-specific antibody was used on immunoblots to detect endogenous FOXM1 protein in N/TERT cells with and without UVB or MG132 treatments. In agreement with the above data, endogenous FOXM1 protein was indeed stabilised by UVB or MG132 treatment (Fig. 1G). However, the level of endogenous FOXM1 detected in N/TERT keratinocytes following UVB irradiation was much lower than that of exogenous FOXM1 suggesting that UVB induced stabilisation of FOXM1 alone is sufficient to explain the increased expression of FOXM1 seen in BCC. In both cases, untreated samples showed very little detectable endogenous FOXM1 protein, consistent with a rapid protein phosphorylation/de-phosphorylation turnover mechanism in cycling cells [23].

### FOXM1B potentiated pro-apoptotic factors in primary NHEK

To understand the possible role of FOXM1 in UVB-induced carcinogenesis, we investigated the levels of various pro-apoptotic/stress-response factors such as p21, p38, p53 and poly(ADP-ribose) polymerase (PARP) in primary NHEK. Protein levels of p21, phospho-p38 MAPK, phospho-p53 (Ser 15) and cleaved PARP were found to be preferentially upregulated in FOXM1B-transduced primary NHEK (Fig. 2A, B) compared to EGFP controls suggesting the existence of oncogenic/replicative stress induced by constitutive FOXM1B expression. NHEK cells expressing FOXM1B showed increased p21 protein level compared to EGFP-expressing cells suggesting the existence of oncogenic/replicative stress induced by constitutive FOXM1B expression. In EGFP expressing cells, p21 proteins were barely detectable at 3 and 6 hours following UVB irradiation, whereas, p21 proteins remained detectable in FOXM1B-expressing cells. Upregulation of p21 is linked to keratinocyte cell cycle arrest prior to the onset of terminal differentiation. p21 is subject to degradation following low doses of UV irradiation, which is a proposed mechanism that allows efficient DNA repair [24-27]. The fact p21 protein levels are not suppressed by maximum induction of FOXM1 24 hours following UVB, suggests that other mechanisms are also regulating p21 stability in primary human keratinocytes. Consistent with our finding, a clear reduction of p21 protein during the first 6 hours after UVB has also been observed in primary human normal and neoplastic keratinocytes [28].

Another pro-apoptotic protein p38 MAPK also showed preferential response to FOXM1B expression. FOXM1B-expressing cells showed increased phosphorylation of p38 MAPK (Thr 180/Tyr182) protein levels compared to EGFP-expressing cells. At all time points following UVB exposure, phospho-p38 MAPK protein level was higher in FOXM1B-expressing cells compared to control cells. p38 MAPK activation is known to respond to oncogenic stress involving the phosphorylation and activation of p53 following UV radiation [29]. Therefore, the upregulation of p38 MAPK in freshly transduced FOXM1 cells provides further evidence of an oncogenic stress response. Similarly, phosphorylation of p53 (Ser 15) following UVB-induced DNA damage is known to enhance apoptosis [29]. Although FOXM1B did not increase phospho-p53 protein level in un-irradiated cells, p53 was preferentially activated by FOXM1B following UVB exposure especially at 6 and 24 hours post-irradiation compared to control cells. The marker for apoptosis PARP also showed preferential response to FOXM1B expression where PARP cleavage were activated in FOXM1B but not in EGFP-expressing cells after 3 hours following UVB irradiation (peaking at 24 hours after UVB). In agreement, PI-FACS analysis showed that upregulation of FOXM1B in NHEK did not induce any cell cycle effects in control cells (without UVB) but FOXM1B expression enhanced (~2.4-fold) accumulation of sub-G1 population following UVB compared to EGFP-expressing cells (Fig. 2C). G1-, S- and G2/M-phase values for EGFP vs FOXM1B cells after UVB are as follows: G1 (48.3% vs 44.2%), S (23.9% vs 25.1%) and G2/M (21.3% vs 15.8%), respectively. Collectively, these results show that in the absence of UVB, FOXM1B upregulation alone induced low-levels of pro-apoptotic factors without triggering cell cycle arrest or cell death. However, following UVB exposure, FOXM1B upregulation triggered DNA-damage checkpoint response genes and sensitised primary NHEK to cell death.

### FOXM1B induces genomic instability in human keratinocytes

**Figure 3:**
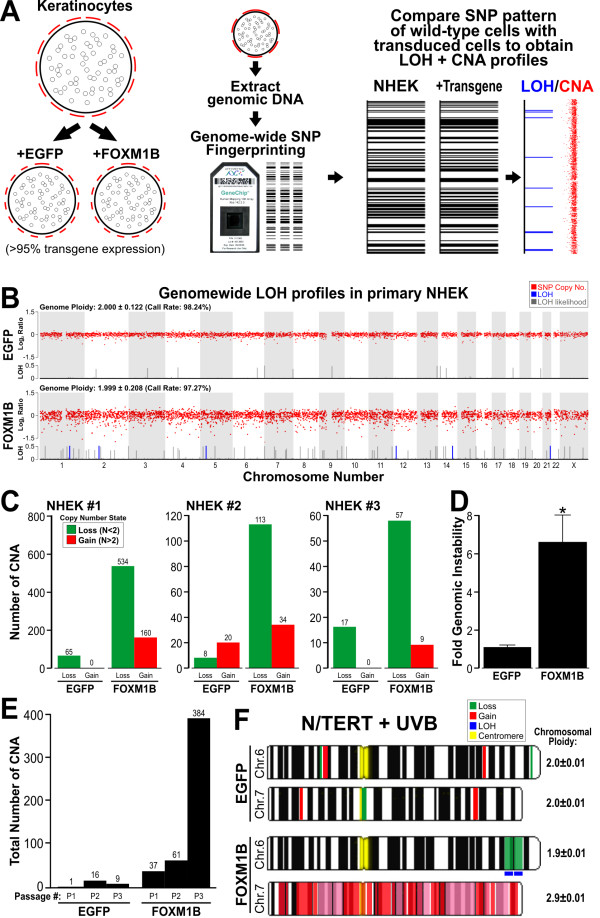
**Acute upregulation of FOXM1B induces genomic instability in primary NHEK**. (A) Early passage (P1) primary NHEK were either mock transduced, EGFP or FOXM1B transduced, left to grow for 4 days and gDNA was harvested for SNP array analysis. LOH and CNV data were obtained by comparing test samples (EGFP or FOXM1B) with reference genome (mock transduced NHEK). (B) CNV (Log 2 ratio, red dots), LOH (blue lines) and LOH likelihood (grey lines) plots for EGFP or FOXM1B expressing cells. LOH likelihood was calculated based on Affymetrix GTYPE algorithm [15,66]. (C) Three normal healthy primary keratinocytes (NHEK#1-3) SNP copy number analysis showing CNV (ploidy number N < 2) and gains (N > 2) in EGFP and FOXM1B overexpressing NHEK, respectively. (D) Average fold-increase in genomic instability of the 3 normal primary NHEK cells in C. *(P < 0.05) indicates significant increase in FOXM1B-induced genomic instability (E) FOXM1B-induced CNV (total SNP number undergoing CNV as indicated above each bar) showed gradual accumulation during a short-term primary NHEK culture (3 passages, P1, P2 and P3). (F) FOXM1B, but not EGFP, enhances LOH and CNV formation in N/TERT cells that survived UVB insult. Ideogram of chromosome 6 and 7 showing regions of CNV and LOH as indicated.

Given that upregulation of FOXM1B triggered various DNA damage/pro-apoptotic stress markers (Fig. 2A, B) in primary NHEK, we hypothesised FOXM1B upregulation could be inducing genomic instability resulting in the upregulation of stress markers. We employed the 10K SNP array to investigate global genomic instability events. Early passage primary NHEK (passage 1) were either mock transduced (no transgene expression) or transduced with either EGFP or EGFP-FOXM1B, left to grow for 4 days and gDNA was harvested for SNP array profiling to obtain genomic instability data in the form of loss of heterozygosity (LOH) and copy number variation (CNV) (Fig. 3A). EGFP upregulation did not induce any detectable LOH or CNV in the NHEK. In contrast, EGFP-FOXM1B upregulation induced a low level but detectable genomic instability where a small number of SNPs had undergone LOH (Fig. 3B, blue lines). Interestingly, FOXM1B-expressing cells showed a ~2-fold increase in LOH likelihood compared to EGFP-expressing cells (Fig. 3B, grey lines). Four days of FOXM1B expression in primary NHEK may not have sufficient time to accrue definitive LOH/CNV loci. This may explain the low number of SNPs acquiring LOH in the FOXM1B expressing cells. We hypothesised that additional/subsequent genomic insults (for example, UVB or chemical carcinogens exposure) to FOXM1B overexpressing cells may expedite the accumulation of oncogenic LOH/CNV loci.

Although FOXM1B did not significantly alter genome ploidy status, CNV (compare red-dot plots in Fig. 3B) appear to have more fluctuations (instability) in FOXM1B (genome ploidy ± sd: 1.999 ± 0.208; SNP Call: 97.27%) compared to EGFP (genome ploidy: 2.000 ± 0.122; SNP Call: 98.24%) expressing cells. In agreement, when examining the copy number of individual SNPs, FOXM1B expressing cells showed ~10-fold increased in CNV (534 losses and 160 gains) compared to EGFP expressing cells showed almost negligible CNV (65 loss and 0 gain). Similar results were obtained from two further independent SNP array experiments with primary NHEK from 2 different normal skin of healthy volunteers (Fig. 3C). The differing degree of FOXM1B-induced genomic instability of the three patients is likely due to individual's variations in intrinsic cellular susceptibility to oncogene expression. Overall, FOXM1B significantly (6.60-fold, p < 0.05, n = 3; Fig. 3D) induced genomic instability in primary NHEK. EGFP upregulation did not induce significant genomic instability. Because of the high sensitivity of SNP array, the genomic instability at such early stage (4 days) following oncogene expression would otherwise be undetectable by other conventional karyotyping methods.

Next, we question whether the acute genomic instability induced by FOXM1B was transient or stable. In a separate experiment, we performed SNP array mapping in NHEK transduced with either EGFP or FOXM1B at three consecutive passages (P1, P2 and P3; Fig. 3E). The SNP data showed that the genomic instability induced by FOXM1B was maintained and accumulated with increasing passage number. EGFP-expressing cells did not show accumulation of genomic instability with increasing passage number. At passage 3, the total number of SNP copy number instability accumulated in FOXM1B-expressing cells (112 losses and 272 gains; total: 384 CNV) was substantially (42.7-fold) higher than in EGFP-expressing cells (0 losses and 9 gains).

### UVB enhances FOXM1B-induced genomic instability

Given the direct induction of genomic instability by FOXM1B in NHEK, and that FOXM1B induced instability in oral mucosal keratinocytes can be augmented by nicotine [10], we were interested to know whether UVB, known to be an etiological factor in BCC formation [18], would also augment genomic instability in primary NHEK. To test this hypothesis, SNP array were performed on UVB-irradiated NHEK cells expressing either EGFP or EGFP-FOXM1B. Unfortunately, following UVB irradiation, primary NHEK (both EGFP and EGFP-FOXM1B expressing cells) underwent terminal differentiation and cell death which did not allow the clonal expansion of UVB resistant cells hence precluding further experiments using primary cells. We therefore performed these experiments using immortalised N/TERT keratinocytes which are more resistant to UVB-induced cell death. Following UVB exposure >95% underwent cell death and the ~5% of surviving cells were allowed to proliferate (~50 days in culture) after which gDNA was harvested for SNP array analyses.

In agreement with our hypothesis, following UVB exposure, cells overexpressing FOXM1B, but not EGFP, showed marked genomic instability especially in chromosome 6 and 7 as illustrated in Fig. 3F. EGFP-expressing cells showed very low levels of random CNV throughout the genome and no LOH was detected. In contrast, FOXM1B-expressing cells showed specific genomic instability in two chromosomes (6 and 7) where a high number of CNV was observed in groups of >16 continuous SNPs. LOH as a result of copy number loss was detected at 6q25.1-6q25.3 (SNP location: 149761596 to 160783097; ~11Mb; see additional file 1), whereas, copy number gain was detected in almost whole of chromosome 7 (7p21.3-7q36.3).

## Discussion

Our previous studies showed that FOXM1B is upregulated in BCC [8] but its role in the tumour initiation remains unclear. The present study investigated the effect of upregulating FOXM1B in primary and immortalised human epidermal keratinocytes. To avoid overexpression artefacts, we titrated retroviral supernatant to achieve levels of FOXM1B expression, similar to those found in various cancer cell lines. This study presents the first evidence that FOXM1B is dose-dependently activated by UVB through protein stabilisation and its upregulation alone induces genomic instability in primary human epidermal keratinocytes.

We found that UVB inhibited proteasomal proteolysis and dose-dependently upregulated FOXM1B protein levels resulting in acute (within 3 hours) FOXM1B protein stabilisation and accumulation in the absence of de novo mRNA/protein synthesis. This agrees with a previous study showing FOXM1 protein stabilisation, rather than de novo mRNA expression, following UV, ionizing irradiation and Etoposide treatment in a human osteosarcoma U2OS cancer cell line [7]. However, it is important to note that whilst UVB was able to upregulate endogenous FOXM1B and that FOXM1 has been shown to induce its own expression [30], other factors such as mutations in PTCH and SMO with subsequent upregulation of Gli transcription factors are most likely to be responsible for the initial upregulation of FOXM1 in BCC ([8]). However, because BCC keratinocytes are very difficult to maintain in culture, it is not possible to investigate this in primary tumour cells. However, the direct activating effect of DNA damage on FOXM1B activity may also explain why genotoxic agents, such as ionising radiation, chemotherapy, intensive photochemotherapy and arsenic intoxication, increase the rate of BCC development [31].

It is known that oncogene expression in normal cells triggers DNA-damage checkpoint as a first anti-cancer barrier response to prevent proliferation of damaged cells [4,32,33]. Our results indicate that acute upregulation of FOXM1B transiently activated CDK inhibitor p21^cip1 ^and stress kinase p38 in primary NHEK. In marked contrast to our study in primary NHEK, Wang et al [34,35] have shown in murine hepatocytes and human U2OS osteosarcoma cells that FOXM1B expression suppressed p21^cip1^ and p27^kip1^ and promoted cell cycle progression. One possible explanation for these differences may be the fact that Wang et al used mouse cells and human carcinoma cells presumably with diverse or abnormal cellular background. In support of this, a recent study investigating the interaction between p53 and FOXM1 showed that different cancer cell lines exhibit different responses to DNA damage-induced FOXM1 levels depending on the p53 expression status [36]. Moreover, we have found that in the N/TERT immortal keratinocyte cell line with suppressed levels of p16^INK4A^ and compromised checkpoint mechanism [11], FOXM1B expression downregulated the levels of p21^cip1^ (data not shown), suggesting a clear difference between primary and cancer cell lines in terms of response to FOXM1B expression. Interestingly, our current study shows that upregulation of FOXM1B in primary NHEK triggered only a minor apoptotic response despite activation of p21^cip1 ^and p38. This suggests that upregulation of FOXM1B allowed cells to tolerate significantly higher levels of p21^cip1 ^and activation of stress kinase p38. Upregulation of FOXM1B in primary NHEK showed enhanced apoptosis following UVB exposure, which is in agreement with a report showing that DNA damage in c-Myc-overexpressing normal mammary epithelial cells, sensitizes cells to DNA damage-induced apoptosis [37]. Despite sensitising cells to UVB-induced apoptosis, the pro-proliferation survival advantage provided by the upregulation of FOXM1B may result in a selection of cells that escape cell death.

The existence of DNA replication stress is a common feature in human pre-cancerous lesions [38] and recently, it has been shown that chronic induction of low, but not high, levels of Ras oncogene activation predisposes cells to tumour formation without inducing permanent cell cycle arrest [39]. Furthermore, a recent study showed that DNA damage upregulates FOXM1 in cells with defective p53 pathway [36]. This may explain our hypothesis that upregulation of FOXM1 following UVB exposure occurs in cells with defective checkpoint mechanism. Therefore, FOXM1 upregulation may provide a mechanism whereby cells evade a checkpoint response which allows damaged cells to proliferate and accumulate genomic instability.

Activation of cellular senescence pathways via the activation of p21^cip1 ^or p16^INK4A ^causes defects in the DNA damage response resulting in increased sensitivity to genotoxic stresses [40]. We propose that FOXM1B-induced activation of p21^cip1 ^or p38 in NHEK may be a result of genomic instability and increase sensitivity to subsequent genotoxic stress (such as UVB) thereby accelerating the selection of genetically unstable cells. We hypothesised that this may be a mechanism whereby upregulation of FOXM1 by UVB may initiate and expedite carcinogenesis.

Given the role of FOXM1B in maintenance of chromosomal segregation and genomic stability [3] and our findings that FOXM1B triggered DNA-damage stress responses (p21^cip1 ^or p38) in primary NHEK following UVB exposure, we investigated whether FOXM1B upregulation may be inducing DNA damage in the form of genomic instability. Recent reports have shown that oncogenes such as Ras induces chromosomal instability to promote malignant transformation [41]. Moreover, we have previously shown that genomic instability was widespread in BCC [14]. We have used a well established and highly sensitive genome-wide Affymetrix SNP mapping technique to profile and quantify genomic instability in the form of LOH and CNV. To our knowledge, this study provides the first evidence that constitutive and acute (4 days) expression of FOXM1B alone in the absent of other stimuli is sufficient to induce LOH and CNV in primary normal human keratinocytes. Furthermore, the FOXM1B-induced genomic instability was accumulated when these cells were passaged in culture. In support of this hypothesis, N/TERT cells expressing FOXM1B, but not EGFP, showed gross chromosomal CNV and LOH following UVB exposure. Interestingly, numerous genes (including MAP3K7IP2, SUMO4, p34/ZC3H12D, LATS1, RAET1 cluster, ULBP cluster, AKAP12, ESR1, MYCT1, VIP, TIAM2, SOD2, WTAP, MAS1, SLC22A cluster, IGF2R, etc.; see see additional file 1) found within the FOXM1B-induced LOH at 6q25.1-6q25.3, have been previously linked to oncogenesis of various human cancers [40,42-53]. Furthermore, in support of our data, genes including EGFR and IGFB1-3 found within the UVB/FOXM1B-induced CNV gain in chromosome 7p12-22 were previously reported to be amplified in HNSCC [54]. This strongly indicates that upregulation of FOXM1B synergises with oncogenic stress (UVB) to promote genomic instability which may help cells gain a survival advantage. In support of our findings in skin keratinocytes, we recently showed that FOXM1B upregulation directly induces genomic instability in primary human oral keratinocytes and that nicotine at a genotoxic concentration promoted FOXM1-induced malignant transformation in oral keratinocytes [10]. Nevertheless, further experiments are required to establish whether the FOXM1B-induced genomic instability is responsible for generating oncogenic LOH and CNV involved in skin malignant transformation.

It is known that FOXM1B plays an important role in the maintenance of genomic stability [3,55] and that FOXM1B is upregulated in majority of human cancers [1]. Although FOXM1B at physiological level has been reported as a regulator of DNA repair [7], its upregulation is likely to interfere with the normal DNA repair mechanism leading to enhanced genomic instability rather than enhanced DNA repair. This highlights the fact that a tight regulation of FOXM1B expression level is required during the cell cycle for proper maintenance of genomic stability. Hence, FOXM1B-induced genomic instability could be a result of aberrant mitotic division due to aberrant expression of mitotic spindle assembly genes such as CEPN-F, Aurora B and Plk1 [3,55] and genes involved in sister chromatids separation and cytokinesis such as CEP55 which we have recently shown to be a downstream target of FOXM1B [10]. In support of our findings, numerous studies have demonstrated that proteins which are important in DNA repair and the maintenance of genomic stability, including mitotic spindle-associated proteins are often found amplified in human cancers, with centrosome amplification being a well characterized mechanism giving rise to genomic instability [56]. Furthermore, consistent with our findings, a recent study has shown that upregulation of FOXM1 cells confer cisplatin resistance in breast cancer cells through deregulation of the DNA repair pathway causing genomic instability [57]. CENP-F (mitosin) overexpression has also been linked to the generation of chromosomal instability in breast cancer patients [58] as well as in head and neck squamous cell carcinomas [59]. Upregulation of Aurora centrosome kinase has been associated with genomic instability in primary human non-small cell lung carcinomas [60], pancreatic cancer [61], and ovarian cancer derived cell lines [62]. Furthermore, FOXM1B upregulation has been reported in majority of human cancers [1], suggesting that gain of FOXM1B function is an important step in human carcinogenesis. In agreement, a recent study measured the levels of aneuploidy, as a marker for genomic instability in 6 different human tumours types, based on genome-wide gene expression pattern, the study found that FOXM1 was the third highest ranked gene with a consensus expression pattern significantly associated with genomic instability in diverse human malignancies [63].

Whilst upregulation of FOXM1B alone can induce genomic instability, we have found that this mechanism alone is not sufficient to induce malignant transformation in NHEK because the rapid replicative exhaustion of NHEK in culture may not allow sufficient time for cells to acquire subsequent oncogenic hits necessary for malignant transformation. In support, FOXM1B overexpression alone did not induce malignant transformation in oral keratinocytes [10]. Indeed, many studies have shown that normal human cells are highly resistant to single-oncogene mediated transformation which usually requires multiple oncogenic hits [64,65]. In line with our findings, in the presence of a second oncogenic pressure such as UVB, FOXM1B, but not EGFP, expressing cells acquired and accumulated definitive LOH and CNV loci, suggesting that upregulation of FOXM1B may predispose cells to malignant transformation. This notion is strongly supported by our previous finding that FOXM1B-expressing oral keratinocytes are highly predisposed to nicotine-induced malignant transformation [10]. Our current study provided further evidence that upregulation of FOXM1B alone without UVB exposure in primary NHEK resulted in genomic instability which could be retained, accumulated and amplified in multiple cell culture passages thereby creating an oncogenic selection pressure prior to UVB exposure.

## Conclusions

This study provided several lines of evidence that FOXM1 protein is accumulated following UVB exposure in normal human skin keratinocytes. Furthermore, we have shown that upregulation of FOXM1B induces genomic instability and potentiated DNA-damage checkpoint responses in primary NHEK following UVB genotoxic stress. However, the subsequent mechanisms of genomic instability and checkpoint responses leading to oncogenesis require further investigation. Nevertheless, we hypothesise that prolonged and repeated UVB exposure selects for skin cells bearing stable FOXM1 protein with aberrant checkpoint may allow ectopic cell cycle entry and subsequent genomic instability. The aberrant upregulation of FOXM1 serves as a 'first hit' where cells acquire genomic instability which in turn predisposes cells to a 'second hit' whereby DNA-damage checkpoint response (eg. inactivation of p53 or p16 or other TSGs) is abolished to allow damaged cells to proliferate and accumulate genetic aberrations/mutations required for cancer initiation.

## Competing interests

The authors declare that they have no competing interests.

## Authors' contributions

MTT conceived, coordinated the study, interpreted data, wrote and finalised the manuscript, performed retroviral transduction, UVB time-course and dose-dependent fluorescence microscopy, FACS, SNP microarray and SNP data analysis. EG performed cell culture, retroviral transduction, immunoblotting, SNP microarray, data interpretation, discussion and manuscript writing and editing. TC and BDY contributed to SNP chip scanning and data interpretation. MPP participated in the design, interpretation and discussion of the study, help editing, writing and finalising the manuscript. All authors read and approved the final manuscript.

## Supplementary Material

Additional file 1 List of genes located within the LOH region of 6q25.1-6q25.3. Click here for file
